# The effect of intravenous lignocaine infusion on intraoperative desflurane requirement: A randomised controlled trial

**DOI:** 10.1097/MD.0000000000044681

**Published:** 2025-10-03

**Authors:** Syarifah Noor Nazihah Sayed Masri, Wilson Anak Matthew Rona, Nurul Alina Muhamad Suhaini, Aliza Mohamad Yusof, Maryam Budiman, Azlina Masdar, Azarinah Izaham

**Affiliations:** a Department of Anaesthesiology and Intensive Care, Faculty of Medicine & Hospital Canselor Tuanku Muhriz, Universiti Kebangsaan Malaysia, Jalan Yaacob Latif, Bandar Tun Razak, Cheras, Kuala Lumpur, Malaysia; b Department of Anaesthesiology and Intensive Care, Hospital Umum Sarawak, Kuching, Sarawak, Malaysia.

**Keywords:** balanced anesthesia, Bispectral Index Scale (BIS), end-tidal desflurane, lignocaine

## Abstract

**Background:**

Perioperative intravenous (IV) lignocaine infusion has a minimum alveolar concentration sparing effect, and this study was designed to investigate the impact of IV lignocaine infusion on the intraoperative end-tidal desflurane (Et-Des) concentration required to maintain the Bispectral Index Scale (BIS) values between 40 and 60.

**Methods:**

Forty-eight patients were recruited for laparoscopic cholecystectomy, appendicectomy, or ovarian cystectomy. They were randomly assigned to Group A, who received a bolus of 1.5 mg kg⁻^1^ of 2% lignocaine hydrochloride over 3 minutes, followed by an IV infusion of 1 mg kg⁻^1^ h⁻^1^ until skin closure, and Group B, who received an equal volume of normal saline. Baseline BIS values, heart rate, and mean arterial pressure were recorded before induction of anesthesia and subsequently every 10 minutes until skin closure. Et-Des concentration, hemodynamic changes, BIS, minimum alveolar concentration, the dose of fentanyl administered, and the amount and cost of desflurane used between the 2 groups were analyzed.

**Results:**

Et-Des concentration in Group A (4.3% ± 0.45%) was significantly lower than in Group B (5.3% ± 0.56%, *P *< .001). The cost of desflurane was significantly reduced in Group A than in Group B (Ringgit Malaysia: 35.79 ± 7.43 vs Ringgit Malaysia: 46.37 ± 9.75, *P* < .001). Both groups’ heart rate and mean arterial pressure showed no significant differences (*P* *=* .484 and 0.619, respectively).

**Conclusion:**

Intravenous lignocaine infusion reduces the Et-Des concentration, amount, and cost required to maintain the BIS value at 40 to 60 without significant hemodynamic changes or side effects in laparoscopic abdominal surgery. The usage of intravenous lignocaine infusion as adjunct to anesthesia is feasible to reduce anesthetic agent requirement.

## 1. Introduction

Lignocaine is an amide local anesthetic and a class 1B antiarrhythmic agent that is widely used in all medical and surgical fields. It also has analgesic, antinociceptive, immuno-modulatory, and anti-inflammatory properties.^[1]^ In addition to its analgesic properties, systemic lignocaine has a minimum alveolar concentration (MAC) sparing effect on volatile anesthetics, proved in multiple animal studies. The mechanism for lignocaine’s MAC limiting impact is likely due to the blockage of sodium channels.^[1]^ A clinical study has shown that intravenous (IV) infusion of lignocaine reduced sevoflurane and propofol requirement during general anesthesia.^[2–5]^ Weinberg et al also tabulated data from numerous trials that detailed the effects of parenteral lignocaine on reducing volatile agent requirements.^[2]^ Furthermore, lignocaine has become an integral component of multimodal anesthesia, contributing to opioid-sparing effects.^[6,7]^ Several studies have shown that perioperative lignocaine infusion significantly reduces intraoperative and postoperative opioid consumption, improves pain scores, and facilitates earlier return of bowel function.^[8,9]^ This opioid-sparing effect is particularly valuable in enhanced recovery after surgery protocols, where minimizing opioid-related side effects is a key goal.

Desflurane is an inhalational anesthetic agent with low blood: gas coefficient (0.42) that allows for more precise control of the level of anesthesia, rapid induction and rapid emergence, resulting in shorter recovery when compared to other inhalational agents such as sevoflurane.^[10]^ Therefore, it is preferably used for long surgery, bariatric surgery, obese patients or elderly patients, as the faster offset leads to an early wake-up, which minimizes the risk of respiratory depression that is commonly associated with these cases.^[11–13]^ As little as 0.02% is metabolized in vivo; hence, no dose adjustment is needed for patients with renal or hepatic dysfunction.^[10]^ However, the cost of desflurane is higher compared to other inhalational agents.^[14]^

Like other volatile agents, cardiovascular depression and the risk of postoperative nausea and vomiting are related to desflurane in a dose-dependent manner.^[15,16]^ Therefore, there is a role for adjunct anesthetic technique such as IV lignocaine to reduce intraoperative requirement of volatile agents. Lower volatile agent requirements should result in a more favorable cardiovascular profile and reduced complications such as nausea, vomiting, and bronchospasm.^[10]^ Furthermore, coupled with opioid reduction, the incidence of postoperative nausea and vomiting could be reduced. Awakening from volatile agent anesthesia is dependent on its washout from alveolar ventilation and its dose at the end of surgery.^[17]^ Therefore, it is postulated that emergence and extubation times might be faster, resulting in more efficient utilization of the operating theater.

Clinical effects of IV lignocaine infusion are widely reported to be seen at 1 to 2 mg kg⁻^1^ h⁻^1^, which results in plasma levels below 5 mcg mL⁻^1^.^[18–20]^ Half-time after a 3-day infusion was approximately about 20 to 40 minutes and there was no accumulation in healthy individuals.^[18]^ Lignocaine has a narrow therapeutic index whereby central nervous system toxicity (numbness of tongue, metallic taste, light headedness, and tinnitus) will begin to manifest when the plasma concentration exceeds 5 mcg mL⁻^1^ and if left untreated, it will progress to visual disturbances, muscle twitching, unconsciousness, seizure, respiratory arrest, and cardiovascular collapse.^[1]^ Cardiovascular toxicity is a rare and late complication as it usually occurs at plasma levels exceeding 10 mcg mL^-1^ which is well above that is required for neurological toxicity.^[21]^ In severe toxicity, intralipid can be administered according to the guidelines provided by the Association of Anesthetists for Great Britain and Ireland.^[22]^

To the best of our knowledge, there is limited research on the effects of IV lignocaine infusion on intraoperative desflurane requirements in the human population.

Severine et al, in their investigation on the effect of lignocaine on postoperative opioid requirements, also found that patients in the lignocaine group required lower doses of desflurane intraoperatively.^[23]^ Similarly, Kuo et al, who compared the effects of thoracic epidural analgesia, IV lignocaine, and placebo on postoperative pain and cytokine response, reported significantly reduced desflurane consumption in both the thoracic epidural and IV lignocaine groups compared to placebo.^[24]^ However, to date, no study has directly compared the effect of IV lignocaine on intraoperative desflurane requirements. Reduction of desflurane requirements when coupled with the reported benefits of IV lignocaine will contribute to a high-quality, cost-effective, optimal, balanced, and safe provision of anesthesia. Our primary objective was to compare the end-tidal desflurane (Et-Des) concentration required to sustain an adequate level of anesthesia by achieving Bispectral Index Scale (BIS) values between 40 and 60 in 48 randomized patients who received IV lignocaine infusion or equal volumes of normal saline during laparoscopic cholecystectomy, appendicectomy or ovarian cystectomy.

## 2. Methodology

This prospective, double-blind, randomized controlled study adhered to the Consolidated Standards of Reporting Trials guidelines and had been approved by the Research Committee Department of Anesthesiology and Intensive Care, Universiti Kebangsaan Malaysia Medical Centre (UKMMC), the Medical Research & Ethics Committee, UKMMC (FF-2021-024) on December 20, 2020, and was registered at ClinicalTrials.gov (NCT06064331) on June 15, 2022. The study was conducted in accordance with the principles outlined in the Declaration of Helsinki.

Written informed consent was obtained from selected patients who were recruited for the study and the study conducted by the principal investigator. This study was conducted at the Hospital Universiti Kebangsaan Malaysia, Kuala Lumpur between January and November 2021. Forty-eight American Society of Anaesthesiology grade I or II patients aged 18 to 75 years with body weights ranging from 50 to 100 kg were enrolled in the study. These patients were scheduled for elective laparoscopic cholecystectomy or hernioplasty, emergency laparoscopic appendicectomy, or ovarian cystectomy with each surgery lasting at least 1 hour. The selected procedures were chosen because they are commonly performed abdominal surgeries associated with moderate levels of surgical stress and postoperative pain. These surgeries typically last at least 1 hour, which ensures sufficient duration for the administration and evaluation of intraoperative interventions such as IV lignocaine. Their inclusion allows for assessment of intraoperative lignocaine’s effects on anesthetic requirement and postoperative outcomes across both elective and emergency surgical contexts, while maintaining clinical homogeneity in terms of surgical invasiveness and expected recovery trajectory.

The exclusion criteria were as follows: American Society of Anaesthesiology ≥ 3, known allergy to the study drug, body mass index > 35 kg m^-2^, use of sedatives, chronic substance abuse, history of seizures, psychiatric disorders, cardiac failure, arrhythmias, and hepatic or renal dysfunction. Patients taking class I B antiarrhythmic drugs or amiodarone were excluded.

Patients were reviewed and recruited during the premedication rounds. Written informed consent was obtained from all patients. The patients fasted for at least 6 hours before surgery. Oral paracetamol 1 g was administered to patients once they were called to the operation theater.

Patients were randomly assigned in a 1:1 ratio into 2 groups (Group A and Group B) using a computer-generated randomization sequence created prior to patient enrollment. The sequence was generated using block randomization with variable block sizes to ensure balanced allocation while preserving allocation concealment. The randomization list was generated by a primary investigator.

To maintain allocation concealment, group assignments were placed in sequentially numbered, opaque, sealed envelopes, which were opened only after patient consent and immediately before drug preparation. Group A received IV lignocaine infusion, while Group B received an equivalent volume of normal saline. The study drugs were prepared by an anesthetist registrar who was not involved in the study’s conduct or outcome assessments. The prepared syringes were identical in appearance, coded according to the randomization sequence, and handed over to the blinded anesthetist incharge for administration. Both patients and anesthetist involved in intraoperative management, postoperative care, and outcome assessment were blinded to group allocation.

In the operation theater, IV access was obtained using an 18 G cannula in all patients, and crystalloid infusion was initiated. Standard monitoring was performed using continuous electrocardiography (ECG), noninvasive BP monitoring, pulse oximetry and capnography. The BIS (BIS Quatro, USA) was used to assess the depth of anesthesia for all patients, and baseline values for the BIS were obtained before induction. Pre-induction BP and heart rate (HR) were recorded. The baseline readings were taken at time 0, indicating the initiation of the bolus drugs. The subsequent data was taken every 10 minutes until skin closure. The study drugs were administered 5 minutes before induction of anesthesia. Group A received an IV bolus dose of 1.5 mg kg^-1^ of 2% lignocaine hydrochloride diluted to 10 mL with normal saline delivered via a syringe pump over 3 minutes followed by maintenance with an IV infusion at 1 mg kg^-1^ h^-1^ of 2% lignocaine hydrochloride in a 20 mL syringe. Patients in Group B received an IV bolus of 10 mL of normal saline over 3 minutes, followed by an IV infusion of 20 mL normal saline.

All patients received 2 μg kg^-1^ IV fentanyl followed by IV propofol of 3 mg kg^-1^ and IV rocuronium 0.6 mg kg^-1^. After 3 minutes of mask ventilation with only 100% oxygen, tracheal intubation using an appropriately sized endotracheal tube was performed to secure the patient’s airway. The patient was then connected to an Aisys CS^2^ (Aisys CS^2^, USA) anesthesia machine, and the lungs were ventilated at a tidal volume of 6 to 8 mL kg^-1^. The end-tidal carbon dioxide level was maintained at between 35 and 45 by adjusting the respiratory rate. The times of induction and intubation were recorded.

Anesthesia was maintained with desflurane in a 50% oxygen–air balance with a total flow of 1 L min^-1^. The Et-Des concentration was adjusted to maintain the target BIS of 40 to 60. The BIS and, MAC values, Et-Des concentration, BP, and HR were recorded every 10 minutes from induction until the end of surgery. IV parecoxib (40 mg) was administered to all patients. Additionally, boluses of IV fentanyl 50 μg were administered to maintain BP within 20% of pre-operative levels. The dosage of fentanyl administered post-induction was recorded. A bolus of IV ephedrine 6 mg was administered during hypotensive episodes with an HR of <60 beats per minute while those with normal HR were administered a bolus of IV phenylephrine 100 mcg. Patients were also administered IV dexamethasone 8 mg post-induction and IV granisetron 1 μg 30 minutes before the end of surgery to reduce the risk of postoperative nausea and vomiting. The core temperatures were maintained above 35°C with a fluid warmer and warming blanket. If surgery lasted more than 3 hours, the patient was dropped out from the study and the data were not analyzed.

At the end of surgery, desflurane and study infusions were discontinued. Neuromuscular block was reversed with a combination of IV atropine 1 mg and IV neostigmine 2.5 mg. When the patient met all criteria for extubation, the endotracheal tube was removed. Time to extubation (defined as the time from desflurane discontinuation to tracheal extubation) was recorded. The total volume and cost of desflurane were automatically calculated by the Aisys CS^2^ (Aisys CS^2^, USA) anesthesia machine and displayed at the end of the case and the data were recorded. The cost was then expressed per minute based on the duration of anesthesia.

Signs of lignocaine toxicity were monitored in all patients from the time of bolus administration of the study drug until 1 hour postoperatively in the recovery bay. During general anesthesia, signs of lignocaine toxicity that were monitored included initial tachycardia and hypertension followed by a prolonged ECG PR segment interval, widened ECG QRS complex, sinus bradycardia, ventricular arrhythmias, hypotension, and cardiac arrest. When the patient was in the recovery bay, along with the late signs of lignocaine toxicity mentioned above, early signs and symptoms characterized by perioral numbness, tinnitus, light headedness, visual disturbances, muscle twitching, seizure, altered mental state, and respiratory arrest were monitored for an hour. If any of these occurred intraoperatively or in the recovery bay, the study drug infusion was stopped, and the patients were dropped out from the study and the data were not analyzed. These patients were managed according to the hospital protocol.

### 2.1. Sample size calculation and statistical analysis

The sample size was calculated using the *t* test by “Power and Sample Size Calculations” software version 3.1.6 (SPSS, IBM, Armonk). The sample size calculation was based on a study by Kuo et al who analyzed cytokine response, postoperative pain and bowel function in patients undergoing colonic surgery after administration of IV lignocaine infusion.^[24]^ Et-Des measurement seen in patients receiving IV lignocaine infusion was 4.0 ± 0.3% versus 4.9 ± 0.2% in the control group. An estimation of the effect size of 0.9 was applied to calculate the sample size. A minimum sample size of 21 patients per group, anticipating a 95% confidence level, and a 10% drop-out rate, resulted in 24 patients per study group.

Data were analyzed using the Statistical Package for the Social Sciences (SPSS) software version 26 (IBM, Armonk). The Kolmogorov–Smirnov test was used to test the normality distribution of the data. Nonnormally distributed demographic and surgical characteristic data were analyzed using the Mann–Whitney *U* test. Normally distributed demographic data were analyzed using the chi-square test. The Student *t* test was used to determine significant differences in continuous variables (Et-Des, BIS, MAC values, haemodynamic parameters, dose of fentanyl used, and amount and cost of desflurane). Continuous data are expressed as mean ± SD. A *P* value <.05 was considered statistically significant.

## 3. Results

**Figure 1.**
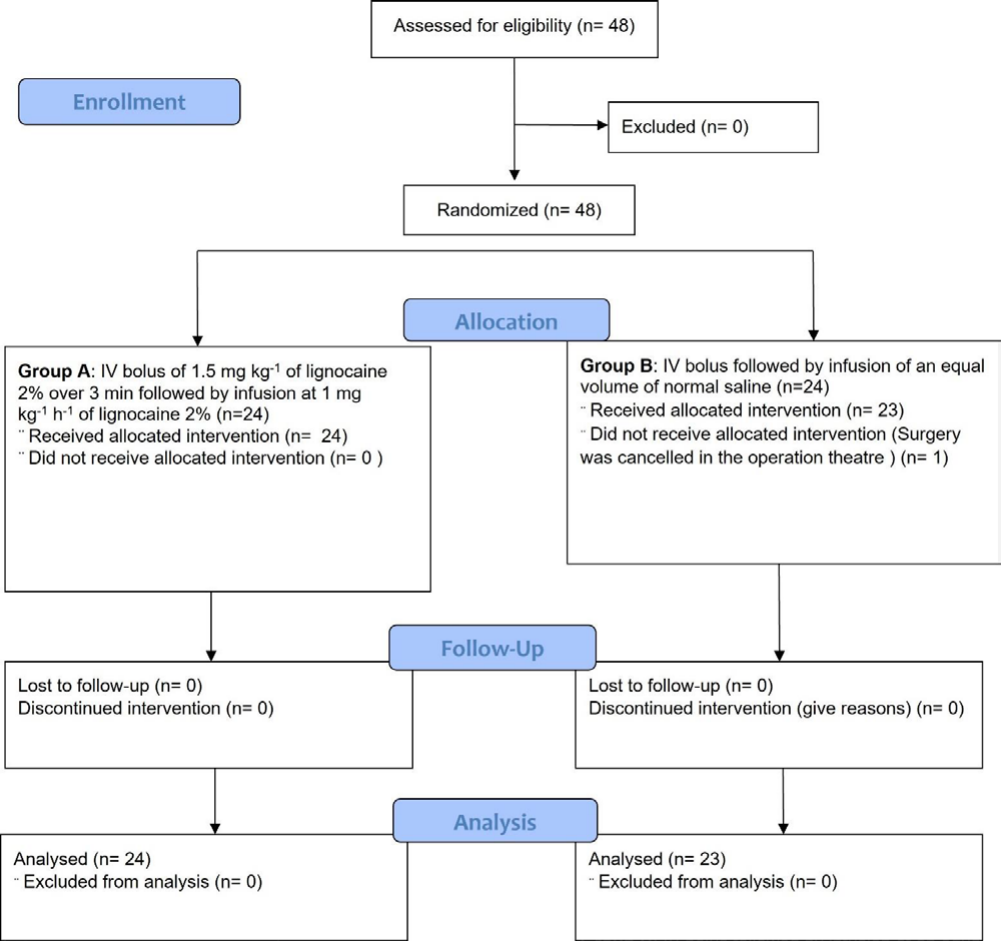
Flow diagram of study patients according to the CONSORT for randomized controlled trials. CONSORT = Consolidated Standards of Reporting Trials.

Forty-eight patients were recruited for the study and randomly assigned to 2 groups, a Consolidated Standards of Reporting Trials diagram as per Figure 1. One patient dropped out of the study because of uncontrolled hypertension with frequent premature ventricular contractions before the induction of anesthesia. None of the surgeries were converted to open procedures.

The demographic data and surgical characteristics of the patients in both groups are shown in Table 1. Demographic data were similar between the 2 groups. The number of cases among the different types of surgeries was also similar (*P* = .180). Additionally, the duration of anesthesia, dosage of IV fentanyl used per kilogram, and extubation times were statistically insignificant between the groups (*P* = .684, .974, and .202, respectively).

**Table 1:** Demographic data and surgical characteristics.

Variable	Group A (n = 24)	Group B (n = 23)	*P* value
Age (yr)	35 ± 18	38 ± 15	.254
Gender			.476
Male	7 (29%)	9 (39%)	
Female	17 (71%)	14 (61%)	
ASA			.476
I	17 (71%)	14 (61%)	
II	7 (29%)	9 (39%)	
BMI (kg m^-2^)	25.9 ± 4.6	24.9 ± 3.9	.438
Type of laparoscopic surgery			.180
Appendicectomy	14 (58%)	19 (83%)	
Cholecystectomy	7 (29%)	3 (13%)	
Ovarian cystectomy	3 (13%)	1 (4%)	
Duration of anesthesia (min)	97.50 ± 32.73	102.17 ± 35.67	.684
IV fentanyl dose (µg/kg)	1.65 ± 1.24	1.63 ± 1.07	.974
Extubation time (min)	4.79 ± 1.22	5.30 ± 1.49	.204

Values are expressed as mean ± SD or number (%).

ASA = American Society of Anaesthesiology, BMI = body mass index.

Intraoperative data for both groups are shown in Table 2. The average Et-Des concentration required to maintain the BIS at 40 to 60 were significantly lower in Group A (4.27 ± 0.45) than in Group B (5.35 ± 0.56) with a *P* value of < .001. A MAC value was also significantly lower in Group A (0.7 ± 0.07) and 0.9 ± 0.10 in Group B with a *P* value of < .001. Consequently, the average cost and amount of desflurane used per hour in Group A were significantly lower than those in Group B (*P* value < .001). BIS readings were comparable in both groups. Average intraoperative hemodynamic variables, including the HR and mean arterial pressure showed no significant differences between the 2 groups.

**Table 2:** Intraoperative data.

Variable	Group A (n = 24)	Group B (n = 23)	*P* value
Et-Des (%)	4.27 ± 0.45	5.35 ± 0.56	<.001
BIS	47 ± 3	47 ± 4	.685
MAC	0.70 ± 0.07	0.90 ± 0.10	<.001
Heart rate (bpm)	75 ± 12	77 ± 9	.484
MAP (mm Hg)	81 ± 7	82 ± 7	.619
Amount of desflurane used (mL h^-1^)	16.1 ± 3.2	20.5 ± 4.6	<.001
Desflurane cost (RM h^-1^)	35.79 ± 7.43	46.37 ± 9.75	<.001

Values are expressed as mean ± SD.

BIS = Bispectral Index Scale, Et-Des = end-tidal desflurane, MAC = minimum alveolar concentration, MAP = mean arterial pressure, RM = Ringgit Malaysia.

**Figure 2.**
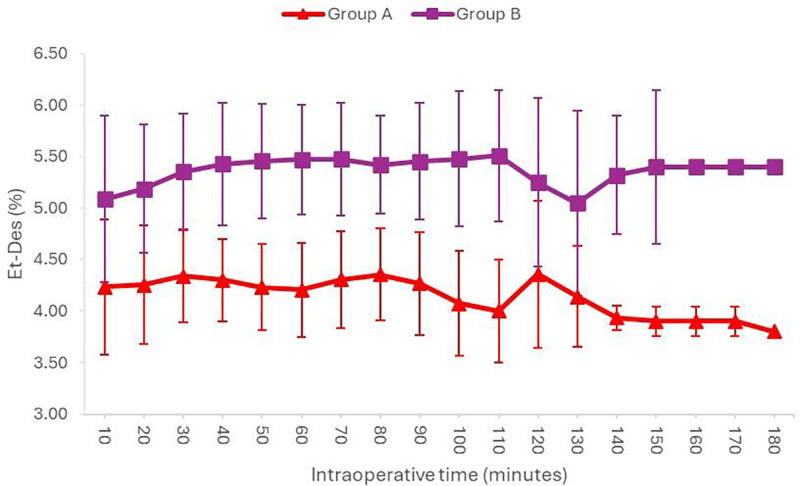
End-tidal desflurane concentration (%) at all intraoperative times.

Figure 2 shows that the intraoperative Et-Des concentrations measured at 10-minute intervals were significantly lower (*P* < .05) in Group A than in Group B at all intraoperative time points.

**Figure 3.**
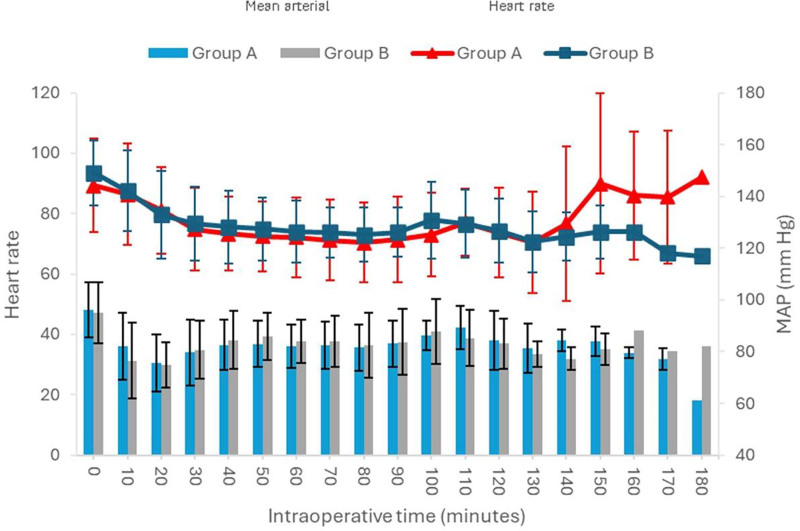
Mean arterial pressure (mm Hg) and mean heart rate (bpm) at all intraoperative times.

Figure 3 showed the intraoperative mean HR and mean arterial pressure measured at 10-minute intervals. The result showed no significant difference of mean arterialpressure and mean HR between the 2 groups at all time intervals.

One patient in Group A developed fast atrial fibrillation with normal BP postoperatively due to hypomagnesaemia in the recovery unit, which resolved after immediate correction with 20 mmol of IV magnesium. No other adverse events associated with systemic lignocaine administration were reported.

## 4. Discussion

The required Et-Des concentration was significantly reduced in patients administered a bolus dose of IV lignocaine 1.5 mg kg^-1^ followed by a constant rate infusion of 1 mg kg^-1^ h^-1^ until skin closure. This finding supported our hypothesis and complemented various studies that which showed that systemic lignocaine has an additive effect with inhalational anesthetic agents to achieve an adequate level of anesthesia.^[2,25]^ Blockage of voltage-gated sodium channels especially in the central nervous system has been postulated to be the primary mechanism by which parenteral lignocaine reduces inhalational anesthetic requirement in a dose-dependent manner.^[26]^

Other studies analyzing the effects of systemic lignocaine as an adjunct to IV general anesthetic such as propofol suggest that it might suppress the uptake of γ-aminobutyric acid which augments γ-aminobutyric acid-mediated chloride channel causing hyperpolarisation of the affected neurons, thereby producing nerve conduction blockade.^[27]^ In addition to these 2 mechanisms, the antinociceptive properties of systemic lignocaine may also be attributed to the inhibition of N-methyl-D-aspartate receptors, thus blunting the surgical stress response which could also play a role in reducing inhalational anesthetic requirement.^[28]^

Hemodynamic parameters, specifically concerning HR and mean arterial pressure, exhibited similarity across both cohorts. This observation aligns with findings from a study conducted by Shafiul et al, which concludes that the group administered lignocaine demonstrated hemodynamic stability.^[29]^ In contrast, other study reported lower BP and HR in patients receiving IV lignocaine infusion.^[2,30]^ These differences could be due to a higher dose of lignocaine used at 1.5 to 2 mg kg^-1^ h^-1^, shorted duration of operation in our study and laparoscopic surgery which was less painful compared to open surgery.

Interestingly, we found that the intraoperative dose of IV fentanyl used in between our 2 groups was comparable. However, some authors have demonstrated that systemic lignocaine infusion is clinically effective in reducing opioid consumption.^[30,31]^ One such study is by Kuo et al, who explored the effects of lignocaine infusion on cytokine response, postoperative pain, and bowel function in sixty randomized patients planned for open colonic cancer surgery.^[24]^ Their study showed that only 1 patient in the group treated with IV lignocaine infusion required additional fentanyl (2 µg kg^-1^) in contrast, 17 out of 20 patients in the control group who were treated with equal volumes of normal saline required 3.3 ± 0.7 µg kg^-1^. This might be due to the higher dose of IV lignocaine used at 2 mg kg^-1^ bolus followed by infusion at 3 mg kg^-1^ h^-1^. Apart from that, patient underwent laparoscopic surgery will have significantly less pain compared to open surgery.^[32]^

A recent meta-analysis by Weibel et al involving 68 clinical trials on the use of perioperative lignocaine infusion for postoperative pain and recovery in adults recommended a bolus dose of 1.5 mg kg^-1^ followed by infusion of 2 mg kg^-1^ h^-1^.^[33]^ Twenty-seven trials with lignocaine infusion doses ranging from 1.5 to 5 mg kg^-1^ h^-1^ reported a small number of patients with mild side effects, such as light headedness or perioral numbness. No incidences of lignocaine toxicity or side effects were reported in our study.

The lower requirement of the Et-Des concentration led to a significant reduction of 21% in the amount used per hour and 23% in terms of the cost incurred per hour. Other proven cost-effective measures of inhalational anesthetic delivery include low-flow anesthesia and automated control of the Et-Des concentration, which were incorporated in our study as part of our standardized anesthetic technique. Most studies have also shown that desflurane led to improved operation theater utilization by faster turnover rate, shorter recovery stays and better Aldrete scores in the post-anesthesia care unit especially in obese patients.^[11–13]^ When integrated with the effect of lignocaine infusion in reducing Et-Des concentration requirement, the cost-saving benefit could theoretically, be substantial.

Our study had several limitations. First, the plasma concentration of lignocaine was not sampled which could provide beneficial data for future studies to determine the optimal dose. Second, we did not consider the use of ultra-low-flow anesthesia which might contribute to a much lower cost and better environmental impact.

## 5. Conclusion

In summary, a bolus dose of 1.5 mg kg^-1^ of IV lignocaine followed by infusion at 1 mg kg^-1^ h^-1^ reduced the Et-Des concentration required and cost per hour without significant intraoperative hemodynamic changes or side effects in patients undergoing laparoscopic abdominal surgery. The usage of IV lignocaine infusion as adjunct to anesthesia is feasible to reduce anesthetic agent requirement.

## Author contributions

**Conceptualization:** Aliza Mohamad Yusof, Maryam Budiman, Azlina Masdar.

**Data curation:** Wilson Anak Matthew Rona, Maryam Budiman.

**Formal analysis:** Wilson Anak Matthew Rona.

**Funding acquisition:** Azlina Masdar.

**Investigation:** Wilson Anak Matthew Rona, Nurul Alina Muhamad Suhaini, Azarinah Izaham.

**Methodology:** Wilson Anak Matthew Rona, Nurul Alina Muhamad Suhaini, Azarinah Izaham.

**Project administration:** Syarifah Noor Nazihah Sayed Masri, Wilson Anak Matthew Rona, Nurul Alina Muhamad Suhaini, Maryam Budiman, Azlina Masdar, Azarinah Izaham.

**Resources:** Aliza Mohamad Yusof, Maryam Budiman, Azlina Masdar, Azarinah Izaham.

**Supervision:** Syarifah Noor Nazihah Sayed Masri, Azlina Masdar.

**Validation:** Aliza Mohamad Yusof.

**Visualization:** Aliza Mohamad Yusof, Maryam Budiman, Azlina Masdar.

**Writing – original draft:** Syarifah Noor Nazihah Sayed Masri, Azlina Masdar.

**Writing – review & editing:** Syarifah Noor Nazihah Sayed Masri, Wilson Anak Matthew Rona, Azlina Masdar.
